# Thymoma and Thymic Carcinoma: Surgical Resection and Multidisciplinary Treatment

**DOI:** 10.3390/cancers15071953

**Published:** 2023-03-24

**Authors:** Yue Zhang, Dong Lin, Beatrice Aramini, Fu Yang, Xi Chen, Xing Wang, Liang Wu, Wei Huang, Jiang Fan

**Affiliations:** 1 Department of Thoracic Surgery, Shanghai General Hospital, Shanghai Jiao Tong University School of Medicine, Shanghai 200025, China; 2 Division of Thoracic Surgery, Department of Medical and Surgical Sciences-DIMEC of the Alma Mater Studiorum, University of Bologna, G.B. Morgagni-L. Pierantoni Hospital, 47121 Forlì, Italy

**Keywords:** thymoma, thymic carcinoma, surgery, multidisciplinary treatment, resectability, subxiphoid thymectomy

## Abstract

**Simple Summary:**

Surgery is the main treatment for thymic tumors, and multidisciplinary treatment would benefit patients a lot. Despite numerous technological advances in the surgical treatment of thymic tumors, a number of contentious issues remain, including the selection of surgical approaches for difficult cases, the selection of Video-assisted Thoracoscopic Surgery approaches, the evaluation of resectability, minimal invasive surgery for locally advanced thymic tumors, lymphadenectomy in thymic tumors, neoadjuvant therapy, debulking surgery, salvage surgery, etc. In solving these problems, the surgeon’s judgment, surgical experience, and surgical skills are especially important; we discuss these debatable issues and share our experience about it which may give some ideas and help to the surgeons working on thymic tumors.

**Abstract:**

Thymoma and thymic carcinoma are the most common tumors of the anterior mediastinum and a relatively rare type of thoracic cancer. The prerequisite for surgery is clinical staging and operative evaluation, both of which are based on medical imaging. The best strategy for treating a thymic epithelial tumor is surgical resection of the organ and surrounding tissue. Thymectomy modalities vary, including open surgery and minimally invasive surgery, and surgeons have used various innovations to better meet the needs of the procedure; therefore, it is critical to select the appropriate procedure based on the patient’s characteristics. Evaluation of resectability is the first step of surgical resection for thymic tumors without distant metastasis. The decision regarding unresectability should be made carefully. During subsequent chemotherapy or chemoradiotherapy, reevaluation of whether an area is resectable or not remains essential. Despite numerous technological advances in the surgical treatment of thymic tumors, several contentious issues remain, including the selection of surgical approaches for difficult cases, the selection of video-assisted thoracoscopic approaches, the evaluation of resectability, minimally invasive surgery for locally advanced thymic tumors, lymphadenectomy in thymic tumors, neoadjuvant therapy for thymic tumors, debulking surgery, and salvage surgery. In solving these problems, the surgeon’s judgment, surgical experience, and surgical skills are especially important.

## 1. Introduction

Thymoma and thymic carcinoma are epithelial tumors that originate from the thymus. They are the most common tumors of the anterior mediastinum and are a relatively rare type of thoracic cancer; they are considered rare diseases, with 1.5 cases per million in the United States of America and 3.93 cases per million in China. Real-world incidence rates are still underestimated. However, in recent years, the number of identified cases of thymoma and thymic carcinoma could be found more frequently because of the wide application of computed tomography [1,2]. Thymoma is the largest subgroup which constitutes 75–80% of thymic epithelial tumors. It is largely inactive, and the patients’ 5-year survival rates are 90% approximately [3,4,5]. While thymic carcinoma is more aggressive and has a worse prognosis, which constitutes 10–20% of TETs, the 5-year survival rates are 55% [6,7,8,9,10,11].

Thymic carcinoma has complex histological subtypes. Thymoma is classified as Type A, Type AB, Type B1/B2/B3, and other rare kinds in the fifth edition of the World Health Organization (WHO) histological classification. The WHO fifth edition classification includes molecular features, such as gene mutations and gene fusions; all of these features contribute to a better understanding of disease biology [12]. Masaoka–Koga staging is based on the extent of invasion [13]. It is closely related to the survival of patients.

Approximately 38% of thymoma patients have myasthenia gravis (MG) compared with no more than 5% of thymic carcinoma and thymic carcinoid patients. MG has a role in the early detection of tumors, and approximately 20% of patients with MG will present with thymoma; however, it is not an independent prognostic factor [14,15]. Surgery may result in great survival and a low recurrence rate, as well as a significant improvement in symptoms and a good neurologic prognosis [16,17].

Thymic tumors are most often detected on physical examination or imaging, following the presence of compression symptoms caused by the compression of surrounding tissues. Contrast enhanced computed tomography (CE-CT) is one of the most popular and useful imaging technologies because it shows the link between the tumor and the surrounding structures and organs. Most importantly, when paired with a surgeon’s skill, CE-CT can detect tumor infiltration symptoms. Genetic, molecular, and multi-omics studies have identified thymoma features that may provide new evidence for thymic tumor prediction, recurrence, and drug treatment. Molecular changes can define more aspects regarding cancer pathogenesis with the possibility to set future targeted treatments [18]. Yamada et al. discovered tuft cell, a new subset of thymic and pulmonary carcinoma which suggests a new mechanism of carcinogenesis and may be related to specific drug susceptibility [19].

Surgical resection is the mainstay of the treatment for thymoma and thymic carcinoma, while a multimodal approach is proved to be useful for many patients [7,20,21,22]. The ability to perform complete surgical resection to treat thymic carcinoma and thymoma is an important factor in recurrence and survival after surgery [23,24]. The surgical treatment of thymic tumors is mainly open surgery; median sternotomy is the most common surgical approach; however, for some complicated cases, the surgical incision is adjusted to a clamshell incision, hemi-clamshell incision, or reverse-T incision. When compared to open surgery, VATS thymectomy and robotic video-assisted thoracoscopic surgery (R-VATS) thymectomy have equivalent if not higher oncological efficacy, more manageable perioperative complications, and better survival outcomes [25,26]. The pathological stage and the radicality of the surgical resections are factors that predict patient survival.

A satisfactory prognosis has been linked to locoregional recurrence and repeated removal of the recurring tumor, and unresectable recurrence of a thymic tumor predicts a poorer prognosis [14,27,28]. Many tumors that were previously considered benign have the potential to metastasize and recur after surgery. For this reason, the surgeon’s assessment of resectability is crucial. A number of controversial issues remain, such as the selection of surgical approach for challenging cases, selection of VATS approach, evaluation of resectability, minimally invasive surgery (MIS) for locally advanced thymic tumors, lymphadenectomy, neoadjuvant therapy, debulking surgery (DS), and salvage surgery (SS). Researchers have also focused on newly emerging methods, such as targeted drugs and immune therapies, although some outcomes are not favorable [29].

This review provides an overview of the evolution of multidisciplinary treatment, especially the surgical treatment of TETs and the implementation of multidisciplinary treatment in addition to our own experience and comments about how to manage the debatable issues in this field.

## 2. Methods

We used the keywords “Thymoma” and “Thymic carcinoma” to search the MEDLINE and PubMed databases for relevant randomized trials and other high-quality studies published between 1 January 1995, and 31 January 2023. We mostly considered publications from the last ten years, but we also included some older high-quality publications.

## 3. Surgical Treatment for Thymic Tumor

Tumor staging should be one of the main factors affecting the selection of surgical treatment. Anatomical and biological characteristics also guide surgical approaches. Thymic tumors usually behave indolently and are located in the anterior mediastinum. The tumor may involve the pericardium, lung hilum, and/or cervicothoracic structures. A satisfying surgical approach should provide adequate exposure to the surgical field and cause minimal surgical injury.

### 3.1. Open Surgery

**Figure 1:**
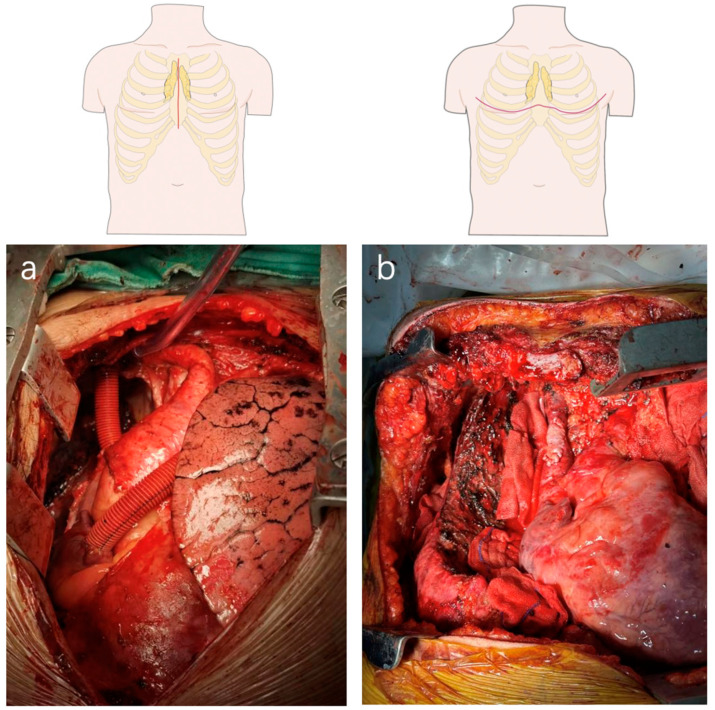
Picture of surgical approach. (**a**). Median sternotomy with a high vascular anastomosis (**b**). Clamshell incision.

The approaches for open surgery are based mainly on the expertise of the surgeon. The optimal approach should provide an adequate surgical view to achieve complete tumor resection and also depends on the surgeon’s preference. The most common surgical approach is represented by a median sternotomy which can present enough exposure of mediastinal structures and provide adequate space for surgical manipulation, especially for the control of great vessels (Figure 1a). However, median sternotomy does not work very well if the pulmonary hilar and other structures of the posterior mediastinum are infiltrated. In these cases, the horizontal incision, hemi-Clamshell, or Clamshell is the first choice (Figure 1b), which allows a more comprehensive view and dissection. Sometimes, these two main approaches are combined in the surgical resection of huge tumors, namely the L-shaped incision and the reversed T-shaped incision. Transcervical incision and lateral incision are also applied in many centers. However, these approaches often do not allow a complete resection of mediastinal tissues due to limited exposure. They are especially not suitable for a thymic tumor with MG.

#### Large Vessel Replacement/CPB or ECMO Assisted Surgery (Agg)

In our center, in terms of open surgery, the clamshell or hemi-clamshell incision is chosen for those difficult cases if:

- Lung hilum(s) is suspected to be involved;
- SVC replacement at a very high level (almost bifurcation of IJV and RSV);
- Synchronous lung cancer surgery requiring standard mediastinal lymphadenectomy;
- Huge tumor impossible to be exposed through median sternotomy;
- Tumor suspicious of invading sternum;
- History of sternotomy.

### 3.2. Minimally Invasive Surgery: New Era and New Challenges

MIS can be divided into many categories depending on the surgical approach, such as transthoracic VATS thymectomy from the right side, VATS thymectomy from the left side, bilateral VATS thymectomy, transcervical VATS thymectomy, subxiphoid thymectomy, which is further divided into CO_2_ inflation type and sternal pull hook elevation type according to the sternal elevation pattern, and robot-assisted thoracic surgery (RATS) thymectomy, which includes transthoracic RATS and subxiphoid RATS (Table 1).

As an emerging technology, MIS has certain advantages, such as three-dimensional high-definition visualization, fully wristed and elbowed instruments, tremor filtration and fatigue avoidance to improve precision and stability, minimal invasiveness with one incision with da Vinci SP, and ICG for nerve visualization to protect nerves [30]. Şehitogullari et al. concluded the robotic and video thoracoscopic approaches are similar for thymoma at stage I and II; however, the combination of robotic-assisted thoracoscopic thymectomy induces advantages postoperatively [31,32]. While high cost and increased dock time are disadvantages compared with other VATS without robots, further studies need to be set to compare long-term follow with the different approaches.

Long-term data regarding the minimally invasive thymectomy are not enough to define the benefits of this surgery compared with the open approach [21]. If all oncologic goals can be met, such procedures may be considered for clinical stages I–II. Despite the perception of a shorter hospital stay and less pain, the advantage of these approaches over open techniques are currently understudied [33]. Since the first application of VATS, surgeons have attempted to use it for thymic tumors. Traditional VATS for thymic tumors are performed through a lateral approach with three ports or a recently popular uniport. RATS also employs a lateral approach. Lateral incision often does not allow a complete resection of mediastinal tissues due to limited exposure; therefore, VATS and RATS with this approach have inherent limitations.

Compared to a unilateral incision, a subxiphoid incision may be a preferable option for VATS. The subxiphoid approach with VATS and sternum retractors is a novel and minimally invasive technique. The patient is in a supine position; a 4 cm vertical incision is performed at 1 cm caudal to the xiphoid process. An additional 2 cm skin incision is made at the level of the fourth intercostal anterior axillary lines on the right side for the thoracoscope placement. The hooks are placed in a 1 cm incision, which was made at the level of the jugular process [34].

#### 3.2.1. Subxiphoid Approach for Better Exposure

The difficulty of mediastinal surgery lies in space issues. The space behind the sternum is narrow, and the distance between the sternum and the heart is small. Due to the presence of the heart, it is difficult to expose both sides of the phrenic nerve and fat at the same time. Compared with VATS, the subxiphoid approach can enter both sides of the mediastinum through one incision after opening the pleura bilaterally [35].

The subxiphoid VATS approach can enable an effective surgical procedure for the entire thymus and bilateral diaphragm nerves. An adequate surgical field of vision is required for the least impactful and most complete thoracoscopy. In 2013, Zielinèski et al. reported that a new sternum double elevation approach (SVT) under a thoracoscopic sword process uses a hook to expand the surgical space by lifting the sternum. It is considered a safe and feasible method. Research has shown that this approach also has a positive effect on the Masaoka stage III thymus [36,37].

Subxiphoid surgery is associated with less bleeding and a shorter hospital stay [38,39,40,41,42,43,44,45]. A meta-analysis revealed that R0 resection has no significant event on the overall R0 resection rate [21]. According to the current literature, MIS had two major advantages: reduced pain after surgery and an improved aesthetic result. The concern of many physicians about MIS is that it may increase the risk of tumor manipulation, which may result in envelope disruption, tumor dissemination, incomplete resection, and local recurrence. Burt et al. conducted a multicenter retrospective cohort study on 943 patients comparing MIT with open thymectomy (OT) and concluded that MIT was not associated with lower rates of R0 resection than OT. Further research into the reasons and long-term data are required [46].

Subxiphoid access is generally achieved through one or two ports, which avoids intercostal nerve damage and other possible incisions. The subxiphoid surgical approach is desirable due to its combination of significant operative field exposure and MIS.

#### 3.2.2. Double Elevation of Sternum for Stage I–III Patients

**Figure 2:**
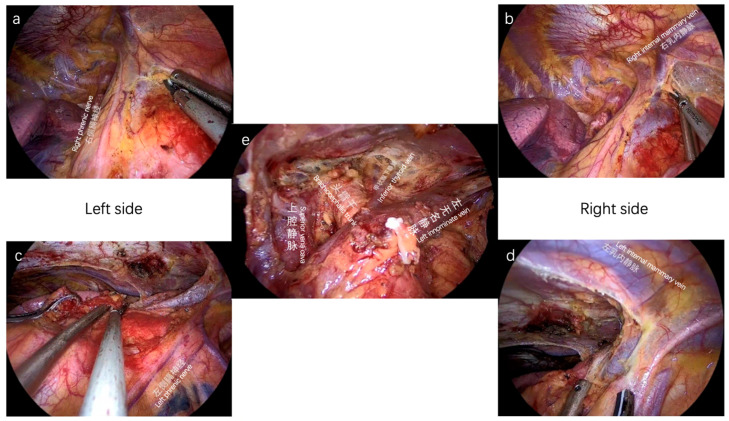
Increased retrosternal space is created by our sternum-lifting system. The relevant anatomy is easily identified, and more complex dissection can be achieved. (**a**) The right phrenic nerve; (**b**) The right internal mammary vein; (**c**) The left phrenic nerve; (**d**) The left internal mammary vein; (**e**) Superior vena cava, brachiocephalic trunk, inferior thyroid vein, left innominate vein.

In our center, the subxiphoid VATS approach with double elevation of sternum by a specially designed lifting system is a routine procedure for the resection of an anterior mediastinal tumor or total thymectomy (Video S1). Compared with intercostal VATS and conventional subxiphoid VATS, our method allows for more elevation of sternum and more retrosternal space. For surgeons, more retrosternal space means more exposure, more dissections, more safety, and more exploration (Figure 2). Our experience suggests that Masaoka stage III thymomas may benefit from thymectomy using the subxiphoid technique with the double elevation of the sternum, and we advise using this strategy as the first line of treatment for prospective Masaoka stage III thymomas [47]. The definition of advanced thymoma is based on pathological specimens presenting infiltration of thoracic structures, such as mediastinal pleura, pericardium, phrenic nerve, lungs, large vessels, etc.) or pleural-pericardial spread [22]. The subxiphoid VATS approach may also be appropriate for treating some locally advanced thymic tumors [48]. Moreover, with the advantages of our method, intraoperative bleeding control from the innominate vein and the vena cava can be managed by Alice forceps and sutures under thoracoscope view [49].

## 4. Debatable Issues about Surgery for Thymic Tumors 

### 4.1. Resectability: Where Is the Border?

Evaluation of resectability is the first step in the surgical resection of thymic tumors without distant metastases. Resectability of locally advanced neoplasms is a highly controversial topic, especially for thymic tumors because the evaluation of resectability is based mainly on the expertise of the surgeon. Due to the complex anatomy of the mediastinum, it is easy to involve surrounding vital structures in a procedure for a locally advanced thymic tumor; this makes the procedure unpredictable and broad. The types of surgery for cases that involve adjacent anatomies to the thymic tumor are listed as follows:

- If a vascular structure is involved, such as a replacement of the vena cava [50] or complicated replacement of the aortic arch and its branches [51];
- If a lung is involved, such as lung resection [52] or extrapleural pneumonectomy [53];
- Specific surgical incisions, such as the common median sternotomy [14] or the clamshell incision [54].

**Figure 3:**
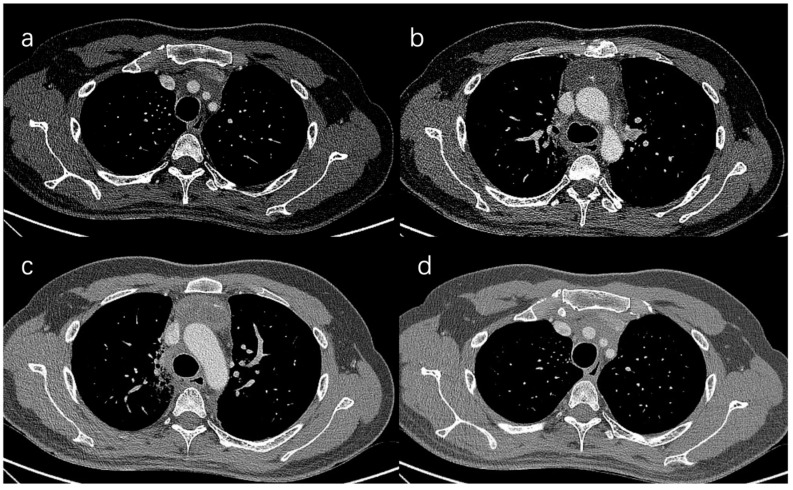
CT scan of 32-year-old patient with thymoma. (**a**) The relationship of tumor to the top of SVC, which is tumor free. The left innominate vein joins the SVC; (**b**) The relationship of tumor and the top of superior vena cava which is tumor free; (**c**) The relationship of tumor and the aorta arch which is potentially tumor free; (**d**) The relationship of tumor and the aorta branches, which are potentially tumor free.

Case introduction: preoperative evaluation reports the left innominate vein and SVC involvement with a definite condition for replacement of the SVC, the aortic arch and its branches uncertain, and the lung uncertain. We conclude that this case is highly likely to be resectable and worthy of exploration, regardless of conflicting decisions by other centers (Figure 3). 

Where is the true border of resection? The standard surgical procedure recommends complete thymectomy, including removal of the thymic tumor, residual thymus, and peri-thymic fat [55]. In surgery for thymic tumors, complete resection usually requires the resection of adjacent structures, such as the pleura, lung, pericardium, phrenic nerve, and even major vessels. For tumors associated with MG, complete resection requires resection of the mediastinal fatty tissues and bilateral pleura, that is, a total thymectomy. In a recent retrospective study, Michelle S. Ginsberg analyzed 41 patients, including all Masaoka stages, to identify features associated with incomplete resection. They concluded that tumor size and infiltration of the vessels predicted incomplete resection and were also present in patients with late Masaoka stage (III/IV), including infiltration of the mediastinal fat [56].

Usually, more radical resections are associated with more curative outcomes; however, these procedures may also be accompanied by increased morbidity, mortality, and medical expenses. The true border is the balance between risks and benefits. Bilateral wedge resection of lung tissues is often acceptable if the pulmonary function is tolerable; in contrast, bilateral phrenic nerve resection is usually unacceptable due to the high risk of severe respiratory morbidity. In thymic surgery, surgeons usually have awareness of unresectability but sometimes neglect potential resectability.

In summary, surgical resectability of an advanced thymic tumor strongly depends on the surgeon and medical team’s experience. We strongly recommend that complex thymoma and thymic carcinoma be treated using multidisciplinary consultation in large, highly specialized centers.

### 4.2. Minimally Invasive Surgery for Locally Advanced Thymic Tumors

MIS, according to ESMO and NCCN guidelines, is an option for early-stage tumors for expert and well-trained surgeons. However, can MIS be used for stage III tumors? With the improvement of VATS techniques, some authors have successfully performed total thymectomy for locally advanced thymoma [57]. In a case of stage III tumor that invaded an innominate vein, Wang et al. reported that the characteristics of the subxiphoid double hook have a good surgical effect on the treatment of vascular invasion for stage III tumors, and complete resection can be achieved [47].

**Figure 4:**
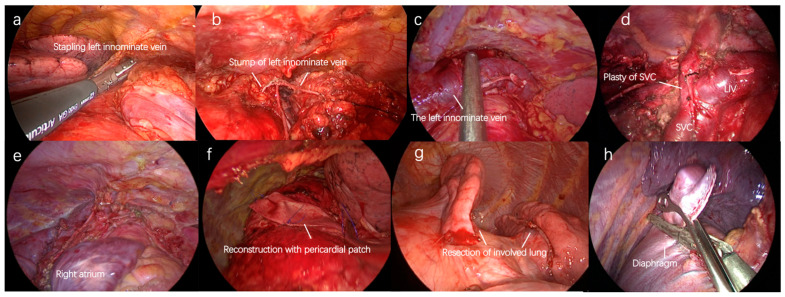
Subxiphoid approach with double sternal elevation for locally advanced thymic tumors. (**a**,**b**) resection of left innominate vein; (**c**) plasty of left innominate vein; (**d**) plasty of SVC; (**e**,**f**) resection and reconstruction of pericardium; (**g**) resection of involved lung; (**h**) prophylactic plication of the diaphragm.

In our center, simultaneous resection of involved lung, plasty or resection of innominate vein, plasty of SVC, and resection or reconstruction of the pericardium can be achieved through the subxiphoid approach with double sternal elevation for locally advanced thymic tumors (Figure 4). Although the outcomes are not completely defined, it is recommended that VATS exploration be attempted first.

### 4.3. Lymphadenectomy in Thymic Tumors

There is limited information on the occurrence, pattern, and prognostic importance of nodal metastases in thymic malignancies, and there is no clear agreement about lymph node dissection. To try to address the issue of lymphadenectomy during surgery, Brascia D et al. conducted a systematic review. They assessed 9452 patients with thymic cancers, including 976 patients with lymph node metastases, and concluded that patients with thymic malignancies should be divided into high- and low-risk groups based on tumor histology, tumor size, and T category. For stage II or higher, WHO histology type B2/3, C, NETTs, and tumor size > 6 cm, systematic lymphadenectomy is advised as it enables radical resection of the tumor and enhanced disease management and PFS [58]. Lymph node dissection can not only increase the reliability of tumor staging, but it also increases disease control and allow facilitates radical extirpation of tumor.

In our center, open total thymectomy is usually performed for N1/2 resection; in VATS, total thymectomy and the subxiphoid approach with double elevation of the sternum are a routine and standard surgical procedure for thymic tumors. Therefore, N1 is automatically resected along with en bloc total thymectomy; however, N2 cases, such as station R 2/4 and L 5/6, are dissected based on intraoperative findings. Preoperative PET-CT is also taken into consideration to direct lymphadenectomy.

### 4.4. Debulking Surgery

Thymic cancers have the highest long-term survival rates. It is controversial whether DS should be undertaken for Masaoka stage III thymoma. The current literature on survival after thymoma DS is contradictory. A meta-analysis of published retrospective cohort studies found stronger evidence for the association of DS with improved overall survival when compared to treatment with surgical biopsy alone [59]. In any case, there is no strong evidence to support the use of DS to treat thymoma.

If we must choose one side, we prefer the role of thymoma DS for the following reasons:

- Thymoma has a much better prognosis due to its slow growth and low rate of distant metastasis;
- DS may reduce tumor size, reducing the harm caused by later radiation therapy to nearby tissue [60];
- Preference for DS leads to more investigation of potentially resectable thymoma.

DS is only considered in our center for unresectable tumors that cannot be removed intraoperatively.

### 4.5. Salvage (Rescue) Surgery (SS)

Salvage surgery is characterized as surgical removal of persistent or recurring primary thymic malignancies after curative local therapies or exclusive chemotherapy in the case of large tumors. The indications and outcomes of SS in thymic malignancies are unknown. Petrella F. and colleagues examined their experience with 21 patients with mediastinal cancers requiring salvage extended mediastinal resection and found that SS might be curative in chosen patients with acceptable morbidity and mortality [61].

Based on the experience from our center, SS is recommended for multidisciplinary-approved cases and should only be performed in highly specialized and experienced centers. In our center, SS is encouraged for cases that were initially assessed as unresectable by a local hospital, have received radical chemoradiotherapy, and have been evaluated as potentially operable. 

## 5. The Role of a Multidisciplinary Team in Surgical Decision Making

Evidence suggests that multimodality treatment of stage III and IV thymic tumors result in satisfactory long-term outcomes; neoadjuvant chemotherapy improves resectability and survival at both stages of the disease [62]. Multidisciplinary treatment improves complete resection outcomes and survival rates.

### 5.1. Oncology Therapy

For TET with an inoperable diagnosis, preoperative chemotherapy or chemotherapy alone can be considered. Preoperative chemotherapy may allow some patients to achieve R0 resection. Cytotoxic medicines continue to play a significant role in the management of thymic malignancies due to a limited understanding of their underlying molecular processes and cellular characteristics. The current standard chemotherapy regimen for thymoma and thymic carcinoma remains unclear due to the lack of randomized controlled studies. Patients with R1 resected stage II–IV thymic carcinoma may be considered for adjuvant chemotherapy postoperatively, especially if they did not receive preoperative induction therapy [47,63].

Chemotherapy alone is the only recommended treatment for metastatic thymomas that are not surgically resectable and not amenable to radiotherapy (stage IV), but it does not benefit stage III–IV completely resected thymomas or thymic carcinomas [64,65]. Advanced or metastatic thymoma and thymic carcinoma can be treated with platinum-based combination chemotherapy, which can shrink the tumor and relieve tumor-related symptoms; however, whether it can prolong survival remains to be determined, but some clinical trials are underway [66].

Several studies have shown that thymic tumor epithelial cells exhibit high programmed cell death-ligand 1 (PD-L1) expression, with PD-L1 expression in thymomas reaching 23–68% [65,67,68,69], suggesting that immune checkpoint inhibitors (ICIs) targeting PD-L1 have some promise for the treatment of thymomas. He et al. described the genomic and transcriptomic profiles of thymic carcinoma samples from 10 patients who received pembrolizumab treatment. They discovered that PD-L1 expression and changes in genes or pathways associated with PD-L1 expression could be potential predictors of immunotherapy response in patients with advanced thymic carcinoma [70]. PD-L1 expression may be a predictive marker for the efficacy of anti-PD-1/PD-L1 monoclonal antibodies in the treatment of thymic carcinoma; however, large-scale phase III clinical studies are still needed to confirm these findings.

Pembrolizumab has been suggested as a promising treatment option for thymic carcinoma. Giaccone et al. completed a single-arm phase-II study of pembrolizumab in patients with recurrent thymic carcinoma; they found that pembrolizumab is active as a second-line therapy in patients with thymic carcinomas, but it was correlated with a higher rate of severe immune-related adverse events (15%) than other types of tumors [71,72]. Therefore, immunotherapy for the management of TETs has significant advantages and disadvantages [73]. The complications related to immunotherapy should be accounted for, especially for patients with autoimmune diseases. Giaccone et al. found that the incidence of autoimmune disorders was higher than expected in a phase-II study of pembrolizumab in patients with advanced thymic carcinoma for whom previous therapies were ineffective [74]. As a result, pembrolizumab is recommended in the guidelines as a second-line therapy in thymic carcinoma but not in thymoma [75]. Immunotherapy patients should be selected carefully, and toxicology monitoring is required. Furthermore, lenvatinib, a new multi-targeted inhibitor of VEGFR, FGFR, RET, c-Kit, and other kinases, also has a positive effect. The REMORA trial reported that lenvatinib has confirmed safety and activity for advanced and metastatic thymic carcinoma (response rate [90%CI, 25.6–52.0, *p* < 0.0001]) [76].

More studies on immune combination therapy or monotherapy are needed because these are potential targets for optimizing therapeutic strategies targeting cancer. In addition, research on the tumor microenvironment of thymic neoplasms is critical from a scientific and clinical standpoint. This research will define the proportion of TETs and the immunophenotype, which represent potential immune checkpoints. Theocharis et al. reported that the affluence and maturation state of various effector components of adaptive immunity were the determining factors for the effective use of immunotherapeutic regimens and the occurrence of autoimmune conditions associated with TETs [77]. Several other ICIs (immune checkpoint inhibitors) are worth observing. Xin Z. et al. conducted an extensive immunophenotyping study of TETs and found that molecular subtypes of tumor cells determined the immune microenvironment of their tumors, where GNB and CHI3L1 may predict the immune function of these tumors and thus the tumor’s behavior [78].

Chemotherapy is an effective approach in cases with low PD-L1 expression. There have also been some attempts at combining chemotherapy and immunotherapy. Yoichi Nishii et al. published the first case report of successful pembrolizumab maintenance therapy in advanced thymic cancer following effective first-line therapy with carboplatin-based chemotherapy with pembrolizumab [79]. The CAVEATT study intends to evaluate the synergistic effectiveness and safety of anti-angiogenesis medicines combined with immune checkpoint inhibitors. They found that avelumab with axitinib offers promise anti-tumor efficacy with tolerable toxicity in patients with advanced type B3 thymoma and thymic carcinoma that has progressed following treatment [80]. Jordi Remon and colleagues’ PECATI trial is phase-II research aimed to assess the effectiveness and safety of lenvatinib plus pembrolizumab in patients with advanced B3-thymoma or thymic cancer who progressed during or after at least one prior course of platinum-based chemotherapy [81]. Aprile V. et al. concluded that hyperthermic intrathoracic chemotherapy (HITHOC) combined with surgery presents better results than surgical treatment alone [82]. These studies may lead to new treatment options for individuals with B3 thymic tumors, which have a poor prognosis and aggressive behavior.

### 5.2. Radiation Therapy

Radiation therapy can be utilized as adjuvant therapy following chemotherapy and surgery for patients with locally advanced cancer, as part of the postoperative management of thymic tumors, and in the management of unresectable malignancies. Patients with unresectable or incompletely resected invasive tumors should receive definitive radiation therapy [83]. Recommendations for radiotherapy require communication between experienced radiation oncologists and surgeons to determine the risk factors. Postoperative radiotherapy (PORT) benefits Masaoka II and Masaoka III patients according to a meta-analysis [29]. Current guidelines recommend radiotherapy dose and fractionation schemes based on the indication of the radiation and the completeness of surgical resection in postoperative cases. The independent prognostic significance or PORT remains unclear. In completely resected Masaoka stage I thymomas, there is prospective evidence to support the avoidance of PORT; however, there is a lack of prospective evidence to guide the use of PORT in other situations. PORT should be administered for thymic carcinoma of any stage with positive resection margins [83]. Several retrospective studies have recommended the consideration of PORT for the advanced stages of disease (stage III–IV), which are in the poor prognostic subset [84]. It is debatable whether PORT is beneficial for all Masaoka stage II thymomas that have been completely resected. There are still many challenges, and there are recommendations to reduce dose volumes to all normal structures [85].

## 6. Discussion

Regarding the diagnosis and treatment of thymic tumors, there are still many controversies and uncertainties. The formation of several groups, such as the ITMIG and other thymic working groups (e.g., ESTS, RHYTMIC), has been beneficial for optimizing the strategies for treating thymic malignancies [86]. There is still a lack of large-scale prospective and randomized clinical trials to obtain high-quality evidence and add practices to the guidelines. The number of patients in each cohort is limited. There are many difficulties with conducting related research. First, a thymic tumor has a low incidence, has multiple subtypes, and lacks unique morphological features and immunophenotypes. Second, a thymic tumor is a relatively indolent tumor; patients may still survive for a long time even after progression and recurrence. Early-stage TET may also recur after complete resection, so the target of increased overall survival is difficult to achieve. Long-term follow-up is needed to further define oncological outcomes. Furthermore, randomized controlled studies are necessary to draw firm conclusions. Due to the distinct characteristics of thymic tumors, another appropriate index to assess clinical efficacy should be observed. The collaboration and communication of cases and approaches between different centers will enhance the learning between doctors and inspire innovation in research. With the combination of disciplines and the integration of superior resources, it will provide standardized and specialized treatment strategies for patients with thymic tumors.

Treatment regimens should be administered jointly by a multidisciplinary team and should also reflect the unique circumstances and preferences of the patient. An increasing number of experts in the field of oncology believe that multidisciplinary treatment can produce for patients can develop better and more comprehensive treatment plans than mono-disciplinary treatment, optimize medical resources, make timely and rapid treatment plans, improve efficiency, reduce the disadvantages of individualism and empiricism, and facilitate the standardization of treatment, which can eventually achieve the effect of 1 + 1 > 2.

## 7. Conclusions

This review provides an overview of the evolvement of multidisciplinary treatment of especially the surgical treatment of thymic epithelial tumors and the implementation of multidisciplinary treatment. Over the past decade, advances in diagnostic techniques, surgical approaches, and systemic therapies for thymic tumors have produced relevant incremental progress in patient outcomes. Resectability status should be determined by a multidisciplinary team after evaluation with high-quality cross-sectional imaging; surgeons’ experiences should also play an important role in this determination. Surgery remains the primary treatment modality to palliate cancer-related symptoms and prolong life. The surgeon’s experience and skill with this type of patient are critical. MIS is more widely implemented now than before. While more prospective collaborative data are required to determine the value of these techniques, advancements in surgical techniques can potentially aid in improving outcomes for patients with complex thymic tumors.

## Figures and Tables

**Table 1:** The Pros and Cons of different surgical approaches of MIS for thymectomy.

Methods	Pros	Cons
Unilateral Transthoracic VATS Thymectomy	May be consistent with the surgical habits of thoracic surgeons	Pain, Injury Insufficient exposure
Transcervical VATS Thymectomy	Less injury	Difficult to resect completely
Conventional subxiphoid VATS Thymectomy	Less pain Better exposure	Insufficient space under the sternum
Subxiphoid VATS Thymectomy with Double elevation of sternum	Raising the sternum sufficiently	Require special equipment

## Supplementary Materials

The following supporting information can be downloaded at: https://www.mdpi.com/article/10.3390/cancers15071953/s1, Video S1: Establishment of sternum lifting system and demonstration of the level of elevation of the sternum.

## References

[1] Engels E.A. (2010). Epidemiology of thymoma and associated malignancies. J. Thorac. Oncol..

[2] Fang W., Fu J., Shen Y., Wei Y., Tan L., Zhang P., Han Y., Chen C., Zhang R., Li Y. (2016). Management of thymic tumors-consensus based on the Chinese Alliance for Research in Thymomas Multi-institutional retrospective studies. J. Thorac. Dis..

[3] Zhao Y., Shi J., Fan L., Hu D., Yang J., Zhao H. (2016). Surgical treatment of thymoma: An 11-year experience with 761 patients. Eur. J. Cardiothorac. Surg..

[4] Huang J., Rizk N.P., Travis W.D., Riely G.J., Park B.J., Bains M.S., Dycoco J., Flores R.M., Downey R.J., Rusch V.W. (2009). Comparison of patterns of relapse in thymic carcinoma and thymoma. J. Thorac. Cardiovasc. Surg..

[5] Masaoka A. (2010). Staging system of thymoma. J. Thorac. Oncol..

[6] Detterbeck F., Youssef S., Ruffini E., Okumura M. (2011). A review of prognostic factors in thymic malignancies. J. Thorac. Oncol..

[7] Detterbeck F.C., Zeeshan A. (2013). Thymoma: Current diagnosis and treatment. Chin. Med. J..

[8] Conforti F., Pala L., Giaccone G., De Pas T. (2020). Thymic epithelial tumors: From biology to treatment. Cancer Treat. Rev..

[9] Rajan A., Zhao C. (2019). Deciphering the biology of thymic epithelial tumors. Mediastinum.

[10] Detterbeck F.C., Parsons A.M. (2004). Thymic tumors. Ann. Thorac. Surg..

[11] Litvak A.M., Woo K., Hayes S., Huang J., Rimner A., Sima C.S., Moreira A.L., Tsukazan M., Riely G.J. (2014). Clinical characteristics and outcomes for patients with thymic carcinoma: Evaluation of Masaoka staging. J. Thorac. Oncol..

[12] Marx A., Chan J.K.C., Chalabreysse L., Dacic S., Detterbeck F., French C.A., Hornick J.L., Inagaki H., Jain D., Lazar A.J. (2022). The 2021 WHO Classification of Tumors of the Thymus and Mediastinum: What Is New in Thymic Epithelial, Germ Cell, and Mesenchymal Tumors?. J. Thorac. Oncol..

[13] Detterbeck F.C., Nicholson A.G., Kondo K., Van Schil P., Moran C. (2011). The Masaoka-Koga stage classification for thymic malignancies: Clarification and definition of terms. J. Thorac. Oncol..

[14] Di Crescenzo V.G., Napolitano F., Panico C., Di Crescenzo R.M., Zeppa P., Vatrella A., Laperuta P. (2017). Surgical approach in thymectomy: Our experience and review of the literature. Int. J. Surg. Case Rep..

[15] Bernard C., Frih H., Pasquet F., Kerever S., Jamilloux Y., Tronc F., Guibert B., Isaac S., Devouassoux M., Chalabreysse L. (2016). Thymoma associated with autoimmune diseases: 85 cases and literature review. Autoimmun. Rev..

[16] Gilhus N.E. (2016). Myasthenia Gravis. N. Engl. J. Med..

[17] Aprile V., Korasidis S., Bacchin D., Petralli G., Petrini I., Ricciardi R., Ambrogi M.C., Lucchi M. (2021). Thymectomy in Myasthenic Patients with Thymoma: Killing Two Birds with One Stone. Ann. Thorac. Surg..

[18] Roden A.C., Ahmad U., Cardillo G., Girard N., Jain D., Marom E.M., Marx A., Moreira A.L., Nicholson A.G., Rajan A. (2022). Thymic Carcinomas-A Concise Multidisciplinary Update on Recent Developments From the Thymic Carcinoma Working Group of the International Thymic Malignancy Interest Group. J. Thorac. Oncol..

[19] Yamada Y., Simon-Keller K., Belharazem-Vitacolonnna D., Bohnenberger H., Kriegsmann M., Kriegsmann K., Hamilton G., Graeter T., Preissler G., Ott G. (2021). A Tuft Cell-Like Signature Is Highly Prevalent in Thymic Squamous Cell Carcinoma and Delineates New Molecular Subsets Among the Major Lung Cancer Histotypes. J. Thorac. Oncol..

[20] Ruffini E., Filosso P.L., Guerrera F., Lausi P., Lyberis P., Oliaro A. (2018). Optimal surgical approach to thymic malignancies: New trends challenging old dogmas. Lung Cancer.

[21] Friedant A.J., Handorf E.A., Su S., Scott W.J. (2016). Minimally Invasive versus Open Thymectomy for Thymic Malignancies: Systematic Review and Meta-Analysis. J. Thorac. Oncol..

[22] Chiappetta M., Grossi U., Sperduti I., Margaritora S., Marulli G., Fiorelli A., Sandri A., Mizuno T., Cusumano G., Hamaji M. (2021). Which Is the Best Treatment in Recurrent Thymoma? A Systematic Review and Meta-Analysis. Cancers.

[23] Kondo K., Monden Y. (2003). Therapy for thymic epithelial tumors: A clinical study of 1320 patients from Japan. Ann. Thorac. Surg..

[24] Hamaji M., Allen M.S., Cassivi S.D., Nichols F.C., Wigle D.A., Deschamps C., Shen K.R. (2012). The role of surgical management in recurrent thymic tumors. Ann. Thorac. Surg..

[25] Agatsuma H., Yoshida K., Yoshino I., Okumura M., Higashiyama M., Suzuki K., Tsuchida M., Usuda J., Niwa H. (2017). Video-Assisted Thoracic Surgery Thymectomy Versus Sternotomy Thymectomy in Patients With Thymoma. Ann. Thorac. Surg..

[26] O’Sullivan K.E., Kreaden U.S., Hebert A.E., Eaton D., Redmond K.C. (2019). A systematic review of robotic versus open and video assisted thoracoscopic surgery (VATS) approaches for thymectomy. Ann. Cardiothorac. Surg..

[27] Rea F., Marulli G., Girardi R., Bortolotti L., Favaretto A., Galligioni A., Sartori F. (2004). Long-term survival and prognostic factors in thymic epithelial tumours. Eur. J. Cardiothorac. Surg..

[28] Yuan Z.Y., Gao S.G., Mu J.W., Xue Q., Mao Y.S., Wang D.L., Zhao J., Gao Y.S., Huang J.F., He J. (2017). Long-term outcomes of 307 patients after complete thymoma resection. Chin. J. Cancer.

[29] Tateishi Y., Horita N., Namkoong H., Enomoto T., Takeda A., Kaneko T. (2021). Postoperative Radiotherapy for Completely Resected Masaoka/Masaoka-Koga Stage II/III Thymoma Improves Overall Survival: An Updated Meta-Analysis of 4746 Patients. J. Thorac. Oncol..

[30] Li F., Ismail M., Elsner A., Uluk D., Bauer G., Meisel A., Rueckert J.C. (2019). Surgical Techniques for Myasthenia Gravis: Robotic-Assisted Thoracoscopic Surgery. Thorac. Surg. Clin..

[31] Sehitogullari A., Nasir A., Anbar R., Erdem K., Bilgin C. (2020). Comparison of perioperative outcomes of videothoracoscopy and robotic surgical techniques in thymoma. Asian J. Surg..

[32] Shen C., Li J., Li J., Che G. (2022). Robot-assisted thoracic surgery versus video-assisted thoracic surgery for treatment of patients with thymoma: A systematic review and meta-analysis. Thorac. Cancer.

[33] Toker A., Sonett J., Zielinski M., Rea F., Tomulescu V., Detterbeck F.C. (2011). Standard terms, definitions, and policies for minimally invasive resection of thymoma. J. Thorac. Oncol..

[34] Aramini B., Fan J. (2019). Technique for Myasthenia Gravis: Subxiphoid Approach. Thorac. Surg. Clin..

[35] Zielinski M., Rybak M., Solarczyk-Bombik K., Wilkojc M., Czajkowski W., Kosinski S., Fryzlewicz E., Nabialek T., Pankowski J. (2017). Subxiphoid thymectomy-technical variants. Video-Assist. Thorac..

[36] Xu H., Liu D., Li Y., Yang L., Wang F., Wang W., Zhang L. (2020). The Outcomes of Subxiphoid Thoracoscopic Versus Video-Assisted Thoracic Surgery for Thymic Diseases. J. Laparoendosc. Adv. Surg. Tech..

[37] Zielinski M., Czajkowski W., Gwozdz P., Nabialek T., Szlubowski A., Pankowski J. (2013). Resection of thymomas with use of the new minimally-invasive technique of extended thymectomy performed through the subxiphoid-right video-thoracoscopic approach with double elevation of the sternum. Eur. J. Cardiothorac. Surg..

[38] Pennathur A., Qureshi I., Schuchert M.J., Dhupar R., Ferson P.F., Gooding W.E., Christie N.A., Gilbert S., Shende M., Awais O. (2011). Comparison of surgical techniques for early-stage thymoma: Feasibility of minimally invasive thymectomy and comparison with open resection. J. Thorac. Cardiovasc. Surg..

[39] Tseng Y.C., Hsieh C.C., Huang H.Y., Huang C.S., Hsu W.H., Huang B.S., Huang M.H., Hsu H.S. (2013). Is thymectomy necessary in nonmyasthenic patients with early thymoma?. J. Thorac. Oncol..

[40] Ye B., Tantai J.C., Ge X.X., Li W., Feng J., Cheng M., Shi J.X., Zhao H. (2014). Surgical techniques for early-stage thymoma: Video-assisted thoracoscopic thymectomy versus transsternal thymectomy. J. Thorac. Cardiovasc. Surg..

[41] Tagawa T., Yamasaki N., Tsuchiya T., Miyazaki T., Morino S., Akamine S., Nagayasu T. (2014). Thoracoscopic versus transsternal resection for early stage thymoma: Long-term outcomes. Surg. Today.

[42] Liu T.J., Lin M.W., Hsieh M.S., Kao M.W., Chen K.C., Chang C.C., Kuo S.W., Huang P.M., Hsu H.H., Chen J.S. (2014). Video-assisted thoracoscopic surgical thymectomy to treat early thymoma: A comparison with the conventional transsternal approach. Ann. Surg. Oncol..

[43] Marulli G., Rea F., Melfi F., Schmid T.A., Ismail M., Fanucchi O., Augustin F., Swierzy M., Di Chiara F., Mussi A. (2012). Robot-aided thoracoscopic thymectomy for early-stage thymoma: A multicenter European study. J. Thorac. Cardiovasc. Surg..

[44] Gu Z.T., Mao T., Chen W.H., Fang W. (2015). Comparison of video-assisted thoracoscopic surgery and median sternotomy approaches for thymic tumor resections at a single institution. Surg. Laparosc. Endosc. Percutan Tech..

[45] Whitson B.A., Andrade R.S., Mitiek M.O., D’Cunha J., Maddaus M.A. (2013). Thoracoscopic thymectomy: Technical pearls to a 21st century approach. J. Thorac. Dis..

[46] Burt B.M., Nguyen D., Groth S.S., Palivela N., Ripley R.T., Makris K.I., Farjah F., Cornwell L., Massarweh N.N. (2019). Utilization of Minimally Invasive Thymectomy and Margin-Negative Resection for Early-Stage Thymoma. Ann. Thorac. Surg..

[47] Wang X., Aramini B., Xu H., Fan J. (2021). Thymectomy with angioplasty through a thoracoscopic subxiphoid approach with double elevation of the sternum in Masaoka stage III thymoma. JTCVS Tech..

[48] Jiang J.H., Gao J., Zhang Y., Wang H., Tan L.J., Ding J.Y. (2021). Modified Subxiphoid Thoracoscopic Thymectomy for Locally Invasive Thymoma. Ann. Thorac. Surg..

[49] Wang X., Aramini B., Zhu Y., Jiang G., Fan J. (2021). Management of bleeding complications during thymectomy by subxiphoid approach with double elevation of the sternum: A case report. Mediastinum.

[50] Maurizi G., Poggi C., D’Andrilli A., Vanni C., Ciccone A.M., Ibrahim M., Andreetti C., Tierno S.M., Venuta F., Rendina E.A. (2019). Superior Vena Cava Replacement for Thymic Malignancies. Ann. Thorac. Surg..

[51] Kuno H., Funaki S., Kimura K., Shimamura K., Kin K., Kuratani T., Sawa Y., Shintani Y. (2019). Complete resection of local advanced thymic carcinoma with total aortic arch replacement after chemotherapy: A case report. Surg. Case Rep..

[52] Hirono M., Nonaka M., Himuro N., Tomita Y., Kataoka D., Kadokura M. (2014). Two cases of thymoma with pulmonary metastasis: A case report. World J. Surg. Oncol..

[53] Wright C.D. (2006). Pleuropneumonectomy for the treatment of Masaoka stage IVA thymoma. Ann. Thorac. Surg..

[54] Aoki K., Izumi Y., Watanabe W., Shimizu Y., Osada H., Honda N., Itoh T., Nakayama M. (2018). Generation of ventilation/perfusion ratio map in surgical patients by dual-energy CT after xenon inhalation and intravenous contrast media. J. Cardiothorac. Surg..

[55] Falkson C.B., Bezjak A., Darling G., Gregg R., Malthaner R., Maziak D.E., Yu E., Smith C.A., McNair S., Ung Y.C. (2009). The management of thymoma: A systematic review and practice guideline. J. Thorac. Oncol..

[56] Hayes S.A., Huang J., Golia Pernicka J., Cunningham J., Zheng J., Moskowitz C.S., Ginsberg M.S. (2018). Radiographic Predictors of Resectability in Thymic Carcinoma. Ann. Thorac. Surg..

[57] Fang W., Feng J., Ji C., Xiang Y. (2016). Minimally invasive thymectomy for locally advanced recurrent thymoma. J. Vis. Surg..

[58] Brascia D., De Palma A., Schiavone M., De Iaco G., Signore F., Panza T., Sampietro D., Di Milo G., Valentini M., Pisconti S. (2021). Lymph Nodes Involvement and Lymphadenectomy in Thymic Tumors: Tentative Answers for Unsolved Questions. Cancers.

[59] Hamaji M., Kojima F., Omasa M., Sozu T., Sato T., Chen F., Sonobe M., Date H. (2015). A meta-analysis of debulking surgery versus surgical biopsy for unresectable thymoma. Eur. J. Cardiothorac. Surg..

[60] Attaran S., McCormack D., Pilling J., Harrison-Phipps K. (2012). Which stages of thymoma benefit from adjuvant chemotherapy post-thymectomy?. Interact. Cardiovasc. Thorac. Surg..

[61] Petrella F., Leo F., Veronesi G., Solli P., Borri A., Galetta D., Gasparri R., Lembo R., Radice D., Scanagatta P. (2008). “Salvage” surgery for primary mediastinal malignancies: Is it worthwhile?. J. Thorac. Oncol..

[62] Lucchi M., Ambrogi M.C., Duranti L., Basolo F., Fontanini G., Angeletti C.A., Mussi A. (2005). Advanced stage thymomas and thymic carcinomas: Results of multimodality treatments. Ann. Thorac. Surg..

[63] Girard N., Ruffini E., Marx A., Faivre-Finn C., Peters S., Committee E.G. (2015). Thymic epithelial tumours: ESMO Clinical Practice Guidelines for diagnosis, treatment and follow-up. Ann. Oncol..

[64] Attaran S., Acharya M., Anderson J.R., Punjabi P.P. (2012). Does surgical debulking for advanced stages of thymoma improve survival?. Interact.Cardiovasc. Thorac. Surg..

[65] Ruffini E., Detterbeck F., Van Raemdonck D., Rocco G., Thomas P., Weder W., Brunelli A., Evangelista A., Venuta F., European Association of Thoracic Surgeons Thymic Working Group (2014). Tumours of the thymus: A cohort study of prognostic factors from the European Society of Thoracic Surgeons database. Eur. J. Cardiothorac. Surg..

[66] Zhang Y., Li Z., Chen Y., Tan L., Zeng Z., Ding J., Du S. (2021). Induction Strategy for Locally Advanced Thymoma. Front. Oncol..

[67] Padda S.K., Riess J.W., Schwartz E.J., Tian L., Kohrt H.E., Neal J.W., West R.B., Wakelee H.A. (2015). Diffuse high intensity PD-L1 staining in thymic epithelial tumors. J. Thorac. Oncol..

[68] Yokoyama S., Miyoshi H., Nishi T., Hashiguchi T., Mitsuoka M., Takamori S., Akagi Y., Kakuma T., Ohshima K. (2016). Clinicopathologic and Prognostic Implications of Programmed Death Ligand 1 Expression in Thymoma. Ann. Thorac. Surg..

[69] Katsuya Y., Fujita Y., Horinouchi H., Ohe Y., Watanabe S., Tsuta K. (2015). Immunohistochemical status of PD-L1 in thymoma and thymic carcinoma. Lung Cancer.

[70] He Y., Ramesh A., Gusev Y., Bhuvaneshwar K., Giaccone G. (2021). Molecular predictors of response to pembrolizumab in thymic carcinoma. Cell Rep. Med..

[71] Cho J., Kim H.S., Ku B.M., Choi Y.L., Cristescu R., Han J., Sun J.M., Lee S.H., Ahn J.S., Park K. (2019). Pembrolizumab for Patients with Refractory or Relapsed Thymic Epithelial Tumor: An Open-Label Phase II Trial. J. Clin. Oncol..

[72] Giaccone G., Kim C., Thompson J., McGuire C., Kallakury B., Chahine J.J., Manning M., Mogg R., Blumenschein W.M., Tan M.T. (2018). Pembrolizumab in patients with thymic carcinoma: A single-arm, single-centre, phase 2 study. Lancet Oncol..

[73] Ballman M., Zhao C., McAdams M.J., Rajan A. (2022). Immunotherapy for Management of Thymic Epithelial Tumors: A Double-Edged Sword. Cancers.

[74] Giaccone G., Kim C. (2021). Durable Response in Patients with Thymic Carcinoma Treated with Pembrolizumab after Prolonged Follow-Up. J. Thorac. Oncol..

[75] Ettinger D.S., Riely G.J., Akerley W., Borghaei H., Chang A.C., Cheney R.T., Chirieac L.R., D’Amico T.A., Demmy T.L., Govindan R. (2013). Thymomas and thymic carcinomas: Clinical Practice Guidelines in Oncology. J. Natl. Compr. Cancer Netw..

[76] Sato J., Satouchi M., Itoh S., Okuma Y., Niho S., Mizugaki H., Murakami H., Fujisaka Y., Kozuki T., Nakamura K. (2020). Lenvatinib in patients with advanced or metastatic thymic carcinoma (REMORA): A multicentre, phase 2 trial. Lancet Oncol..

[77] Masaoutis C., Palamaris K., Kokkali S., Levidou G., Theocharis S. (2022). Unraveling the Immune Microenvironment of Thymic Epithelial Tumors: Implications for Autoimmunity and Treatment. Int. J. Mol. Sci..

[78] Xin Z., Lin M., Hao Z., Chen D., Chen Y., Chen X., Xu X., Li J., Wu D., Chai Y. (2022). The immune landscape of human thymic epithelial tumors. Nat. Commun..

[79] Nishii Y., Furuhashi K., Ito K., Sakaguchi T., Suzuki Y., Fujiwara K., Yasuma T., Kobayashi T., D’Alessandro-Gabazza C.N., Gabazza E.C. (2022). Good Response of Advanced Thymic Carcinoma with Low PD-L1 Expression to Chemotherapy plus Pembrolizumab as First-Line Therapy and to Pembrolizumab as Maintenance Therapy: A Case Report. Pharmaceuticals.

[80] Conforti F., Zucali P.A., Pala L., Catania C., Bagnardi V., Sala I., Della Vigna P., Perrino M., Zagami P., Corti C. (2022). Avelumab plus axitinib in unresectable or metastatic type B3 thymomas and thymic carcinomas (CAVEATT): A single-arm, multicentre, phase 2 trial. Lancet Oncol..

[81] Remon J., Girard N., Novello S., de Castro J., Bigay-Game L., Bernabe R., Greillier L., Mosquera J., Cousin S., Juan O. (2022). PECATI: A Multicentric, Open-Label, Single-Arm Phase II Study to Evaluate the Efficacy and Safety of Pembrolizumab and Lenvatinib in Pretreated B3-Thymoma and Thymic Carcinoma Patients. Clin. Lung Cancer.

[82] Aprile V., Bacchin D., Korasidis S., Nesti A., Marrama E., Ricciardi R., Petrini I., Ambrogi M.C., Paladini P., Lucchi M. (2020). Surgical treatment of pleural recurrence of thymoma: Is hyperthermic intrathoracic chemotherapy worthwhile?. Interact. Cardiovasc. Thorac. Surg..

[83] Gomez D., Komaki R., Yu J., Ikushima H., Bezjak A. (2011). Radiation therapy definitions and reporting guidelines for thymic malignancies. J. Thorac. Oncol..

[84] Lim Y.J., Kim H.J., Wu H.G. (2015). Role of Postoperative Radiotherapy in Nonlocalized Thymoma: Propensity-Matched Analysis of Surveillance, Epidemiology, and End Results Database. J. Thorac. Oncol..

[85] Liu J., Govindarajan A., Williams T.M., Kim J., Erhunmwunsee L., Raz D., Massarelli E., Salgia R., Chen Y.J., Amini A. (2022). An Updated Review on Radiation Treatment Management in Thymus Cancers. Clin. Lung Cancer.

[86] Drevet G., Collaud S., Tronc F., Girard N., Maury J.M. (2019). Optimal management of thymic malignancies: Current perspectives. Cancer Manag. Res..

